# Supplementary material for: Identification and functional analysis of non-coding regulatory small RNA FenSr3 in Bacillus amyloliquefaciens LPB-18
Source: PeerJ. 2023 May 15;11:e15236. doi: 10.7717/peerj.15236 (PMC10194069; doi:10.7717/peerj.15236)
Supplement: Supplemental Information 4 [file peerj-11-15236-s004.zip › KO/CK-vs-T1_map/map05206.html]

KEGG PATHWAY: MicroRNAs in cancer - Reference pathway


|  |  |
| --- | --- |
| **MicroRNAs in cancer - Reference pathway** |  |

[
Pathway menu
| Organism menu
| Pathway entry
| Show description
| User data mapping
]

MicroRNA (miRNA) is a cluster of small non-encoding RNA molecules of 21 - 23 nucleotides in length, which controls gene expression post-transcriptionally either via the degradation of target mRNAs or the inhibition of protein translation. Using high-throughput profiling, dysregulation of miRNAs has been widely observed in different stages of cancer. The upregulation (overexpression) of specific miRNAs could lead to the repression of tumor suppressor gene expression, and conversely the downregulation of specific miRNAs could result in an increase of oncogene expression; both these situations induce subsequent malignant effects on cell proliferation, differentiation, and apoptosis that lead to tumor growth and progress. The miRNA signatures of cancer observed in various studies differ significantly. These inconsistencies occur due to the differences in the study populations and methodologies used. This pathway map shows the summarized results from various studies in 9 cancers, each of which is presented in a review article.

|  |  |  |
| --- | --- | --- |
| Reference pathway | 184% 150% 122% 100% 82% 67% 55% | 图片下载 |
